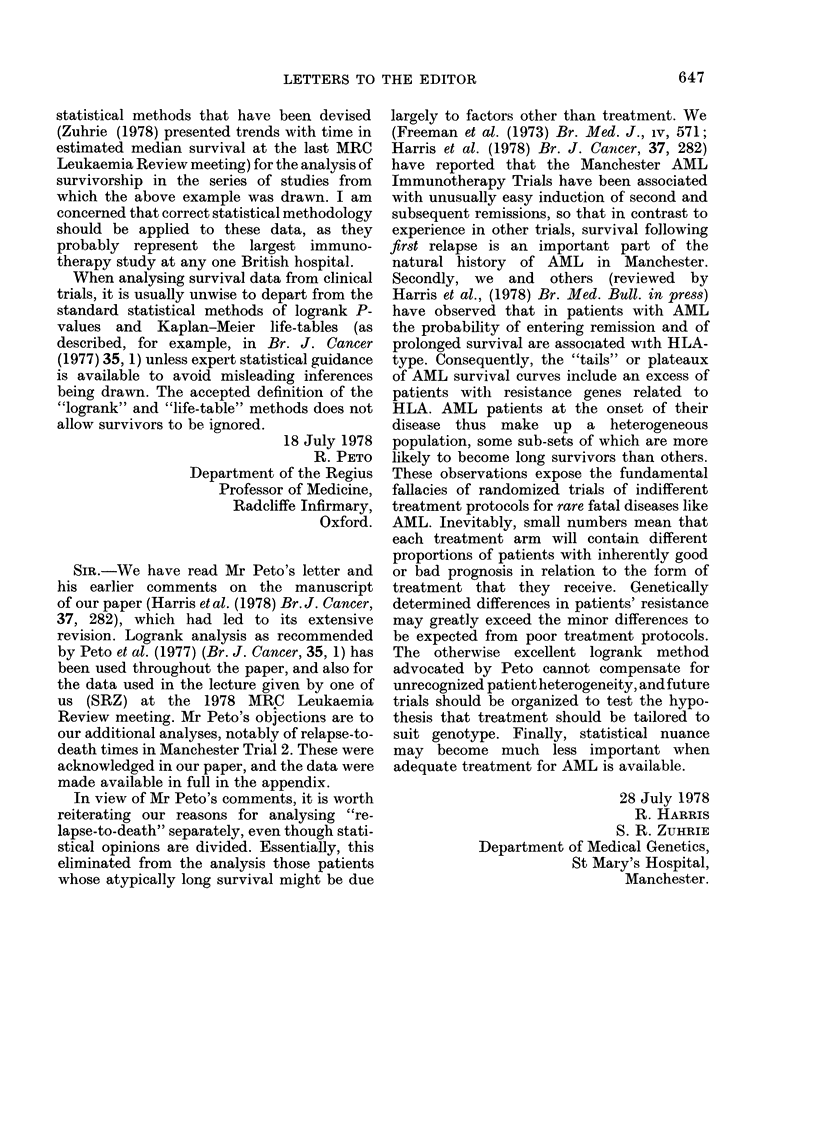# Misleading analyses

**Published:** 1978-11

**Authors:** R. Harris, S. R. Zuhrie


Sir.—We have read Mr Peto's letter and his earlier comments on the manuscript of our paper (Harris *et al.* (1978) *Br. J. Cancer*, **37**, 282), which had led to its extensive revision. Logrank analysis as recommended by Peto *et al.* (1977) (*Br. J. Cancer*, **35**, 1) has been used throughout the paper, and also for the data used in the lecture given by one of us (SRZ) at the 1978 MRC Leukaemia Review meeting. Mr Peto's objections are to our additional analyses, notably of relapse-to-death times in Manchester Trial 2. These were acknowledged in our paper, and the data were made available in full in the appendix.

In view of Mr Peto's comments, it is worth reiterating our reasons for analysing "relapse-to-death" separately, even though statistical opinions are divided. Essentially, this eliminated from the analysis those patients whose atypically long survival might be due largely to factors other than treatment. We (Freeman *et al.* (1973) *Br. Med. J.*, iv, 571; Harris *et al.* (1978) *Br. J. Cancer*, **37**, 282) have reported that the Manchester AML Immunotherapy Trials have been associated with unusually easy induction of second and subsequent remissions, so that in contrast to experience in other trials, survival following *first* relapse is an important part of the natural history of AML in Manchester. Secondly, we and others (reviewed by Harris *et al.*, (1978) *Br. Med. Bull. in press*) have observed that in patients with AML the probability of entering remission and of prolonged survival are associated with HLA-type. Consequently, the "tails" or plateaux of AML survival curves include an excess of patients with resistance genes related to HLA. AML patients at the onset of their disease thus make up a heterogeneous population, some sub-sets of which are more likely to become long survivors than others. These observations expose the fundamental fallacies of randomized trials of indifferent treatment protocols for *rare* fatal diseases like AML. Inevitably, small numbers mean that each treatment arm will contain different proportions of patients with inherently good or bad prognosis in relation to the form of treatment that they receive. Genetically determined differences in patients' resistance may greatly exceed the minor differences to be expected from poor treatment protocols. The otherwise excellent logrank method advocated by Peto cannot compensate for unrecognized patient heterogeneity, and future trials should be organized to test the hypothesis that treatment should be tailored to suit genotype. Finally, statistical nuance may become much less important when adequate treatment for AML is available.

28 July 1978

R. Harris
S. R. Zuhrie
Department of Medical Genetics,
St Mary's Hospital,
Manchester.